# The role of patient navigators in ambulatory care: overview of systematic reviews

**DOI:** 10.1186/s12913-021-07140-6

**Published:** 2021-10-28

**Authors:** Hannah Budde, Gemma A. Williams, Juliane Winkelmann, Laura Pfirter, Claudia B. Maier

**Affiliations:** 1 London School of Economics and Political Science, Houghton St, London, WC2A 2AE UK; 2 European Observatory on Health Systems and Policies, London School of Economics and Political Science, Houghton Street, London, WC2A 2AE UK; 3 Department of Healthcare Management, Technische Universität Berlin, Straße des 17. Juni 135, 10623 Berlin, Germany; 4 Maecenata Institut für Philanthropie und Zivilgesellschaft in Berlin, Rungestr. 17, D-10179 Berlin, Germany

**Keywords:** Patient navigator, Effectiveness, Systematic review, Overview of reviews, Access, Quality, Coordination of care

## Abstract

**Background:**

Patient navigators have been introduced across various countries to enable timely access to healthcare services and to ensure completion of diagnosis and follow-up of care. There is an increasing evidence on the the role of patient navigation for patients and healthcare systems. The aim of this study was to analyse the evidence on patient navigation interventions in ambulatory care and to evaluate their effects on individuals and health system outcomes.

**Methods:**

An overview of reviews was conducted, following a prespecified protocol. All patients in ambulatory care or transitional care setting were included in this review as long as it was related to the role of patient navigators. The study analysed patient navigators covering a wide range of health professionals such as physicians, nurses, pharmacists, social workers and lay health workers or community-based workers with no or very limited training. Studies including patient-related measures and health system-related outcomes were eligible for inclusion. A rigorous search was performed in multiple data bases. After reaching a high inter-rater agreement of 0.86, title and abstract screening was independently performed. Of an initial 14,248 search results and an additional 62 articles identified through the snowballing approach, a total of 7159 hits were eligible for title/abstract screening. 679  articles were included for full-text screening.

**Results:**

Eleven systematic reviews were included covering various patient navigation intervention in cancer care, disease screening, transitional care and for various chronic conditions and multimorbidity. Nine systematic reviews primarily tailored services to ethnic minorities or other disadvantaged groups. Patient navigators performed tasks such as providing education and counselling, translations, home visits, outreach, scheduling of appointments and follow-up. Eight reviews identified positive outcomes in expanding access to care, in particular for vulnerable patient groups. Two reviews on patient navigation in transitional care reported improved patient outcomes, hospital readmission rates and mixed evidence on quality of life and emergency department visits. Two reviews demonstrated improved patient outcomes for persons with various chronic conditions and multimorbidity.

**Conclusions:**

Patient navigators were shown to expand access to screenings and health services for vulnerable patients or population groups with chronic conditions who tend to underuse health services.

**Supplementary Information:**

The online version contains supplementary material available at 10.1186/s12913-021-07140-6.

## Background

The rising prevalence of chronic diseases is a challenge for health systems worldwide. Almost one in three people have been shown to live with one or more chronic conditions across the OECD countries [1]. Strengthening primary care and the coordination of healthcare across inpatient and outpatient services and multiple providers has therefore become essential to deliver high-quality and personalized care to patients [2–5].

Access to healthcare services is vital to meet patients’ needs, decrease health inequalities and prevent diseases or slow disease progression and the development of complications. Yet, throughout their care pathway, many patients see themselves confronted with a fragmented and complex healthcare system and navigating through it poses a challenge [6]. To enhance access to health care and strengthen coordination and continuity of care, various countries have introduced new professional roles and tasks, such as patient navigator roles [7]. The role of patient navigators was first introduced in the United States (US) in the 1990s. The aim was to improve access to cancer care services for minority groups by improving screening and diagnosis of certain types of cancer and assisting patients in manoeuvring through the healthcare system [8].

Patient navigators have been introduced across various countries to enable timely access to healthcare services and to ensure completion of diagnosis and follow-up of care [9–11]. Originally focused on cancer, the role and function of patient navigators have diversified. People with chronic conditions often require repeated contact with multiple health care providers and may experience barriers in accessing healthcare services [12]. In transitional care, earlier discharges from the inpatient to the outpatient and community setting have resulted in expanded and new roles of healthcare workers in the ambulatory care setting, ensuring greater coordination of care and follow-up [13]. Patient navigation may cover various tasks along the care continuum including education, outreach, facilitating communication and end-of-life care [14]. Patient navigator roles are performed by various health professions. They may also be performed by lay persons and peers. The literature shows that this is often the case for patient navigators in cancer care, who closely collaborate with licensed healthcare professionals [11, 15, 16]. Also qualified health professionals such as nurses or social workers are being implemented as patient navigators [17].

Patient navigators are often targeted towards patients from vulnerable or marginalized populations that frequently experience the largest barriers to accessing health care. These include ethnic minorities, older people, socioeconomically disadvantaged groups or uninsured persons [18].

There is an increasing amount of evidence on the positive effect of patient navigation for patients; for instance, on disease prevention and health promotion [19, 20]. There are also various studies with non-significant findings or mixed results [21]. The number of systematic reviews has increased over the past decade, requiring an update of the evidence on the role of patient navigators in different countries and health system contexts, population groups and for various outcomes. To the best of our knowledge, there has been no overview of systematic reviews on the impact of patient navigation on patient and health system outcomes.

The overarching aim of this study was to assess the availability of evidence on patient navigation interventions in ambulatory care and to evaluate their effects on patients and healthcare systems. This study seeks to inform researchers and policy-makers about the relevance and effectiveness of patient navigation.

## Methods

This study was part of a larger study consisting of an overview of systematic reviews on skill-mix changes in ambulatory care. It followed a prespecified protocol (registered with International Prospective Register of Systematic Reviews, [22]) and is described according to the Preferred Reporting Items for Systematic Reviews and Meta-Analyses (PRISMA) statement. An overview of review was chosen due to the high number of expected published systematic reviews on the topic and to identify gaps in the existing literature.

### Eligibility criteria

Eligible systematic reviews or meta analyses were included if they assessed the effect of patient navigator roles on patients or health systems and were published since 2010. The start date reflects our focus on more recent evidence and the increasing interest in patient navigation in the last decade. Only English language publications were included. Protocols of reviews were excluded. Systematic reviews not covering any individual or health-system related outcome measure were also excluded. Detailed eligibility criteria and the search process are outlined in the registered protocol [22].

The overview of reviews followed the Population, Intervention, Comparison and Outcomes (PICO) scheme:

#### Population

All patients and participants in ambulatory care or transitional care (hospital to ambulatory sector) setting were included in this review as long as it was primarily related to the role of patient navigators. Reviews that covered patient navigators in hospital settings only (with no cross-sectoral coordination function described) or emergency care were excluded. The geographic focus was on high-income countries. Hence, systematic reviews explicitly excluding high-income countries (e.g. only including low-income countries) were not considered for inclusion.

#### Intervention

The overview of reviews analysed all roles of patient navigators or similar roles in the country specific contexts. We covered a wide range of persons and health professionals as patient navigators, such as physicians, nurses, pharmacists, social workers and others if performing patient navigator roles. Lay health workers or community-based workers with no or very limited training were also included if they performed patient navigation.

#### Comparison

Standard of care followed the definitions provided by the individual studies included in the systematic reviews.

#### Outcome measures

We included a range of primary outcomes in the overview of reviews. Studies including patient-related measures (e.g. clinical outcomes, mortality, patient satisfaction, quality of life) and health system-related outcomes (access, continuity of care, costs, efficiency) were eligible for inclusion.

### Search method for identification of reviews

#### Search strategy

The search strategy was built and run in Embase first, then adapted to the following databases: Medline in Ovid, Cochrane CENTRAL, Web of Science Core Collection, CINAHL EBSCOhost (Cumulative Index to Nursing and Allied Health Literature), PsychINFO Ovid and Google scholar. A librarian supported the team in developing the search strategy and carried out the literature search in January 2018 and an update in July 2021. Search terms included combined Medical Subject Headings (MeSH) with free text words. For each of the databases, the search strategy was adapted to meet the specific requirements. Filters were used (as applicable, depending on the database) to identify systematic reviews. The search strategy was reviewed internally.

In addition to the electronic search, a snowballing approach was used to detect other systematic reviews. Reference lists of included reviews were screened for other relevant studies and systematic reviews that were identified during the piloting phases were also considered for full-text screening.

### Data collection and analysis

#### Screening of reviews

**Fig. 1:**
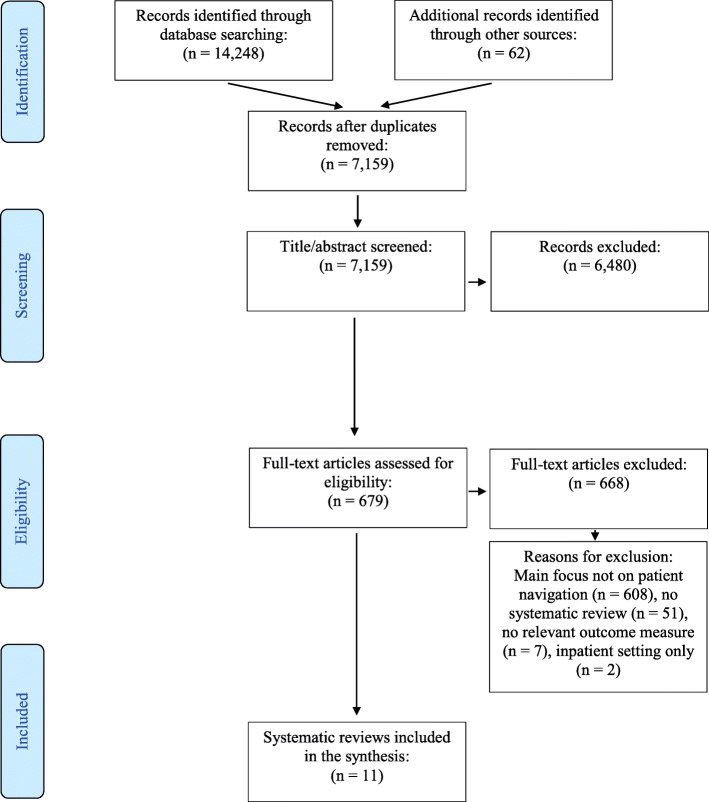
PRISMA Flow Diagram

A total of 14,248 hits were generated by the search and an additional 62 articles were identified through the snowballing approach. After removing all duplicates, the final total included 7159 hits. Three reviewers independently screened titles and abstracts of the first 100 hits, using the software Rayyan QCRI. Inter-rater agreement was calculated using an extended version of Cohen’s kappa coefficient,^1^ and a high (0.86) interrater agreement was reached [23, 24]. The final total of 7159 hits generated by the search and the snowballing approach were screened for title/abstract by the three reviewers. Overall, a total of 679 articles were identified as eligible for full-text screening during the title/abstract screening. These were accessed as full-text and independently reviewed for final inclusion by the researchers, after interrater agreement of 0.78 was reached. Finally, 11 reviews were identified as eligible for final inclusion. Systematic reviews ruled out for inclusion did either not primarily focus on patient navigators, not meet the criteria of being a systematic review, not include any relevant outcome measure or only covered interventions in the inpatient setting (see Fig. 1).

### Data extraction and analysis

A data extraction form (Microsoft Excel) was used, informed by previous overview of reviews [25, 26]. To ensure consistency in data extraction, a piloting phase was performed among the researchers wherein differences were resolved via discussions and one researcher double checked data extraction for all reviews.

The data analysis was performed as narrative synthesis. Meta-analysis was not possible due to the heterogeneity of the outcome measures. The data were summarized for navigation roles focusing on cancer care (diagnosis and treatment), screening of diseases, transitional care and chronic conditions and multimorbidity. Moreover, study design, participants, professions, comparator, country, outcomes (by individual patient outcomes and health system outcomes) were extracted.

### Quality appraisal

Quality appraisel was performed using the AMSTAR II (A MeaSurement Tool to Assess systematic Reviews) checklist. The AMSTAR I checklist was specifically developed to assess the quality of systematic reviews [25], further developed and expanded into AMSTAR II covering a total of 16 items [27]. The systematic reviews were independently evaluated by two researchers after a pilot phase to ensure consistency in the ratings.

## Results

### Characteristics of the reviews included

A total of 11 systematic reviews describing 311 individual studies on patient navigation roles met the inclusion criteria (see Tables 1, 2, 3 and 4). The included reviews covered four areas of care: five systematic reviews analysed patient navigator roles for patients with various types of cancer (‘cancer care’ including diagnosis and treatment), six reviews focused on screenings for the prevention and early identification of diseases, two reviews covered transitional care interventions and two reviews included patient navigator interventions for various chronic conditions and multimorbidity. The analysis of the results is structured along these four areas of care. Patient navigators performed tasks such as providing education and counselling (addressing the language needs of the target groups), translations, home visits, outreach, scheduling of appointments and follow-ups.

Nine of the 11  reviews covered interventions targeted primarily or exclusively at vulnerable population groups such as ethnic minorities, non-native speakers or medically underserved populations [11, 12, 15, 16, 21, 28–30, 33]. Most individual studies were conducted in the US and Canada, followed by studies from European countries (e.g. Austria, Italy, UK), Asia (e.g. Bangladesh, Korea, Japan) and South Africa. Randomized Controlled Trials (RCTs) made up a majority of studies included in the systematic reviews. Two out of the 11 systematic reviews performed meta analyses [15, 32].

All systematic reviews reported on the professional background of the patient navigator (e.g. nurses, social workers, lay health workers). Three systematic reviews provided information on the length or contents of their patient navigation training [21, 29, 30]. The reporting of education and training in the three reviews was often limited to individual studies and not consistent. Reporting on the details of the interventions and outcome measures was available for all systematic reviews.

### Quality appraisal

The quality of the systematic reviews included in the overview of reviews varied. Three were assessed as moderate quality [12, 21, 32] and eight systematic reviews were of low quality [11, 15, 16, 28–31, 33]. Common quality issues related to the description of inclusion criteria, selection of study design or reporting of reasons for exclusion. The full assessment can be found in the supplementary material.

### Patient navigation interventions with a focus on diagnosis and treatment of cancer

Five systematic reviews focused on patient navigation for cancer care covering interventions to improve cancer diagnosis and treatment (Table 1: [11, 12, 16, 21, 28]). Three reviews also included interventions for early diagnosis and screening [11, 12, 16]. The main interventions undertaken by patient navigators were education on the disease, its treatment and self-care, scheduling and assisting with appointments. Other tasks included facilitating communication between providers [11, 12, 16, 21, 28].

**Table 1:** Patient navigator interventions with a focus on the diagnosis and treatment of cancer

*Intervention*				*Outcomes*		
*Details of the intervention*	*Profession(s)*	*Population*	*Countries*	*Patient-related outcomes*	*Health-system related outcomes*	*Source*
Patient navigation included facilitating communication with providers, outreach, assistance with appointments and scheduling, education, follow-up, counselling	Intervention: Patient, nurse, professional navigator Comparison: Radiologists, physicians, breast surgeons	Cancer patients receiving care in ambulatory setting (incl. ethnic minority and minority women patients)	CA, KR, US	• Improved patient satisfaction in four out of four studies, statistically insignificant	• Care coordination improved, statistically insignificant • Shorter time to diagnosis, statistically insignificant	• [21]
Introduction of patient navigator to overcome obstacles such as language barriers, coordination of appointments, lack of transportation and insurance or difficulties to understand the follow-up process	Intervention: Lay persons, nurses with oncology experience, individual with master in social work Comparison: Not reported	Cancer patients from medically underserved populations, rural or urban area, uninsured persons, non-English speaking persons	US	• Improved adherence to follow-up	• Earlier treatment and treatment initiation • Significant improvements in diagnostic resolution	• [28]
Patient navigator intervention to improve screening, diagnosis and treatment of cancer in ethnic minority patients (e.g. scheduling appointments, outreach, assistance with transportation, telephone support)	Intervention: Nurses, lay health educators, lay health workers, NPs, community health aides, physicians Comparison: Not reported	Ethnic minority cancer patients	US	• Improved adherence to screening	• Increased screening rates • Improved completion of screening	• [16]
Patient navigation in breast cancer care involving non-health professionals (e.g. follow-up to screening and clinical breast abnormalities, education, counselling, referral, translation and scheduling)	Intervention: Breast cancer survivors, lay community health workers, nurse navigator in cooperation with lay navigator and social worker, lay workers Comparison: Professions not defined	Breast cancer patients (66% of sample were non-white women)	CA, US	• Improved adherence to breast screening and diagnostic follow-up (e.g. after abnormal radiographic screening, attending genetic counselling)	• Reduced waiting time for biopsy/diagnostic intervals • Decreased time to appointment with genetic counsellor	• [11]
Patient navigation interventions (in person or via phone) focussing on diagnosis and treatment of various types of cancer (e.g. barrier assessment, appointment scheduling, reminders, emotional support, education, liaison with providers)	Intervention: Lay persons, peers, professional workers Comparison: Not reported	Patients with abnormal cancer screening results including mostly vulnerable patients	US, AU	• Increased diagnostic resolution • Improved adherence to follow-up appointments	• Improved time to diagnosis	• [12]

Country abbreviations: *AU* Australia, *CA* Canada, *KR* Republic of Korea, *US* United States of America

#### Professions

Patient navigator roles were undertaken by individuals with diverse backgrounds, ranging from health professionals to lay persons [11, 12, 16, 21, 28] and patients such as breast cancer survivors [11]. Among qualified health professionals, nurses were the most common profession to take on the role of patient navigation. The reporting of details of the training and education of the patient navigators was limited among the five reviews. Only one systematic review reported on patient navigators’ training. In the review, one out of four studies provided details on education, with patient navigators trained in breast health education, public speaking and observing mammograms undertaken in mobile breast cancer screening units by radiologists, breast surgeons and oncologists [21].

#### Population

Out of the five reviews, one targeted ethnic minorities [16] and three covered all cancer patients, of which the majority were ethnic minorities [11, 12, 21]. One review covered medically underserved patients including uninsured persons and patients from rural and urban areas in the US [28].

#### Outcomes

Patient navigation showed improved outcomes in all five systematic reviews focusing on facilitating access to health services [11, 12, 16, 21, 28]. Two systematic reviews demonstrated improvements in access and timeliness of treatment and care for vulnerable patients or ethnic minority patients, for example by reducing waiting times and improving appointment scheduling with specialists [11, 28]. Earlier treatment and treatment initiation were demonstrated by one systematic review [28]. Two reviews showed improved adherence to follow-up for medically underserved patients [12, 28]. Two reviews reported shorter time to diagnosis [12, 21]. One review indicated improved patient satisfaction and coordination of care. However, the authors report that the effect was not statistically significant. This was largely attributed to small sample sizes for sub groups analyses in the evaluated studies [21].

### Patient navigation interventions with a focus on screening of diseases

Six systematic reviews analysed patient navigator roles to increase screening uptake (see Table 2: [11, 12, 15, 16, 29, 30]). Except for one review [15], all reviews focussed on cancer. Three reviews focused on diagnosis and treatment in addition to screening and have therefore also been covered in the previous sub-section [11, 12, 16]. Interventions covered various components such as education, reminders, assistance with appointments, language support and the identification and removal of barriers.

**Table 2:** Patient navigator interventions with a focus on screenings of diseases

*Skill-mix interventions*				*Outcomes*		
*Details of the intervention*	*Profession(s)*	*Population*	*Countries*	*Patient-related outcomes*	*Health-system related outcomes*	*Source*
Patient navigation interventions in person or via phone focussing on screening uptake (e.g. barrier assessment, appointment scheduling, emotional support, education)	Intervention: Lay persons, peers, professional workers Comparison: Not reported	Patients eligible for cancer screening	US, CA, FR	• Improved completion of screening		[12]^a^
Patient navigator interventions either as face-to-face, mail or phone interventions (e.g. education or support in identifying barriers, setting up appointments and making reminder calls)	Intervention: Trained lay-persons or health professionals (e.g. nurse) Comparison: Control group without PN or intervention group before intervention	Patients in primary care completing screening for colorectal, cervical and breast cancers and hepatitis B (often vulnerable patients)	BD, CA, US	• Increased probability to attend recommended care events (OR 2.48, 95% CI, 1.27 to 5.10, *p* = 0.008)	• Increased access to screenings (OR: 2.48, 95% CI, 1.93 to 3.18, *p* < 0.00001)	[15]
Patient navigator intervention to improve screening, diagnosis and treatment of cancer in ethnic minority patients (e.g. scheduling appointments, outreach, assistance with transportation, telephone support)	Intervention: Nurses, lay health educators, lay health workers, NPs, community health aides, physicians Comparison: Not reported	Ethnic minority cancer patients	US	• Improved adherence to screening	• Increased screening rates • Improved completion of screening	[16]^a^
Patient navigation in breast cancer care involving non-health professionals (e.g. follow-up to screening and clinical breast abnormalities, education, counselling, referral, translation and scheduling)	Intervention: Breast cancer survivors, lay community health workers, nurse navigators in cooperation with lay navigators and social workers, lay persons Comparison: Professions not defined	Breast cancer patients (66% of sample were non-white women)	CA, US	• Improved adherence to breast screening and diagnostic follow-up (e.g. after abnormal radiographic screening, attending genetic counselling)	• Reduced waiting time for biopsy/diagnostic intervals • Decreased time to appointment with genetic counsellor	[11]^a^
Patient navigator interventions targeting screening and diagnosis of cancer (e.g. partnerships with health and non-healthcare services, education, scheduling, outreach, communication, follow-ups)	Intervention: Lay health advisors, promotora, case managers, community outreach specialists, lay health educators, lay health worker), partners (e.g. academic researchers, community members, community activists, public health officials) Comparison: Not reported	Medically underserved population (incl. Urban cities, rural counties, suburban neighbourhoods, border regions), screenings for breast, cervical and colorectal cancer	US	• Improved completion of diagnostics, especially for patients who missed a follow-up diagnostic appointment • Improved referral and follow up	• Improved breast, cervical, or colorectal cancer screening rates for mammography, pap tests, screening with colonoscopy • Shorter time to diagnosis for abnormal screening results	[29]
Patient navigation included assisting patients in navigating through cancer screening (e.g. setting up appointments and making reminder calls along with providing language services such as interpreting and one-to one educational sessions)	Intervention: Patient navigators, bilingual staff, health educators, family members, professional interpreters Comparison: Not reported	Non-proficient English-speaking population groups in need for cancer care	US		• Significant increased screenings rates for breast, cervical, or colorectal cancer (14/15 studies) • Breast cancer screening rates increased by 17–25% • Colorectal screening rates increased by 13–40% • Cervical cancer screening showed a nearly 60% increase	[30]

*CI* Confidence Interval, *OR* Odds Rati, *p p*-value, ^a^systematic reviews covering screening, diagnostic and treatment and therefore listed twice; Country abbreviations: *CA* Canada, *BD* Bangladesh, *FR* France, *US* United States of America, promotora = lay health workers of a community (mostly female) providing a range of services as liaison between Hispanic communities and healthcare services

#### Professions

Qualified health professionals [12, 15] or trained lay persons [12, 15, 29, 30] undertook patient navigator roles. In one review, lay patient navigators received general training in information related to cancer and health, cancer screening and guidelines. Moreover, they were trained in providing patient support and care. Skill-based training was provided on topics such as motivational interviewing and communication [29]. In another review, 5 out of 15 studies reported on the length of training of patient navigators. This ranged from 6 hours training to 2 days workshops and additional follow-ups 1 year later [30].

#### Population

Systematic reviews included patients in primary care undergoing screening for cancer [11, 12, 16, 29, 30] or various diseases [15]. Five out of the six reviews included vulnerable patients, such as medically underserved groups [29] or non-English proficient persons [30].

#### Outcomes

Patient navigation improved screening rates for population groups in all six systematic reviews, with the majority of patients being from ethnic minorities [11, 12, 15, 16, 29, 30]. The meta-analysis showed a significant increase in screenings rates with patient navigation (OR: 2.48, 95% CI, 1.93 to 3.18, *p* < 0.001) [15]. Four other systematic reviews also found improved screening rates [12, 16, 29, 30]. One review showed improved adherence to screening for ethnic minority cancer patients [16]. Another systematic review demonstrated improved adherence to breast cancer screening and diagnostic follow up for breast cancer patients, of which the majority were ethnic minority women [11].

Patient navigation also significantly improved the probability of attending recommended care events (OR 2.48, 95% CI, 1.27 to 5.10, *p* = 0.008) [15]. Improved completion of diagnostic tests [29] and screening [12, 16] was also shown. Improved referrals and shorter time to diagnosis for patients with abnormal screening results were reported in one systematic review [29].

### Patient navigator interventions in transitional care

Transitional care interventions from hospital to ambulatory care or home involving patient navigator roles were the focus of two systematic reviews (see Table 3: [31, 32]). Although a wide range of different interventions were covered, patient navigator tasks mostly included care coordination, discharge planning and follow up in addition to education and multiprofessional collaboration among health professionals.

**Table 3:** Patient navigator interventions with a focus on transitional care

*Skill-mix interventions*				*Outcomes*		
*Details of the intervention*	*Profession(s)*	*Population*	*Countries*	*Patient-related outcomes*	*Health-system related outcomes*	*Source*
Patient navigation in transitional care (e.g. discharge planning, coordination of care, phone support, home visits, liaison with medical and community services, patient/caregiver education)	Intervention: Registered nurse with advanced practice expertise, gerontological advanced practice nurse, social worker; physicians and physician mentors (supporter in a multidisciplinary team) Comparison: Not reported	Older patients with chronic diseases	AU, CA, US	• Improved depression symptoms • Improvement for disease self- management • Improved quality of life • Improved activities of daily living, communication with patients, caregivers, education for caregivers, self-management, knowledge of patient medication	• Lower readmissions • Shorter time to readmission and less hospital days • Improved community referrals • Inconclusive effect on costs related to use of emergency rooms	[31]
Transitional care interventions from hospital to home (majority of interventions focused on contacts, home visits and educational components; others included multidisciplinary coordination and collaborations)	Intervention: Nurses, primary care physicians, cardiologists, pharmacist Comparison: Family physician, not consistently reported	Older patients with at least one chronic disease	AT, AU, CA, BE, CH, CN, DE, DK, ES, FI, HK, IR, IT, JP, NL, NZ, SE, SI, UK	• Mortality: Significantly lower for intervention (RD − 0.02, − 0.05-0.00, NNT 50) and maintained at 6, 12 and 18 months post-discharge • No significant effect on quality of life	• Significantly fewer ED visits at 3 months post-discharge (Risk Difference (RD) -0.08, − 0.15, − 0.01); no effect at 1, 6, 12 months • Total readmission days: Significant difference at 3 months (MD − 1.33, − 2.15, − 0.52), 6 months (MD − 1.42, − 2.33, − 0.50), 12 months (MD − 3.37, − 5.21, − 1.53), 18 months (MD − 3.16, − 5.68, − 0.64); no difference at 1 month • Risk hospital admission: Significantly lower at 6 months (RD − 0.05, − 0.09, − 0.00), 12 months (RD − 0.11, − 0.17, − 0.05), and at 24 months (MD − 1.03, − 1.81, − 0.24)	[32]

*CI* Confidence Interval, *ED* Emergency Department, *MD* Mean Difference, *OR* Odds Ratio, *p p*-value, *RD* Risk Difference, Country abbreviations: *AU* Australia, *AT* Austria, *BE* Belgium, *CA* Canada, *CH* Switzerland, *CN* China, *DE* Germany, *DK* Denmark, *ES* Spain, *FI* Finland, *HK* Hoch Kong, *IR* Iran, *IT* Italy, *JP* Japan, *NL* The Netherlands, *NZ* New Zealand, *SE* Sweden, *SI* Slovenia, *UK* United Kingdom, *US* United States of America

#### Professions

Nurses (e.g. advanced practice nurses), physicians, pharmacists and social workers performed patient navigation interventions, among other professions [31, 32].

#### Population

Patient navigation in transitional care focused on older patients with at least one chronic condition [31, 32].

#### Outcomes

Patient navigation in transitional care demonstrated a significant reduction in mortality rates (Risk Difference (RD) -0.02, 95% CI: − 0.05-0.00) [32], improvements for depression symptoms and disease management [31] and a positive effect on activities of daily living (ADL), communication with patients, caregivers, education for caregivers, self-management and knowledge of patient medication [31]. Mixed results on quality of life were reported in the two reviews. No difference in quality of life between intervention and non-intervention groups was reported by one review [32], but improved quality of life reported for the intervention group in the other review [31].

One review demonstrated improved referrals [31]. The other review showed that introducing patient navigation in transitional care results in significantly fewer Emergency Department visits at 3 months post-discharge (RD -0.08, − 0.15, − 0.01). Yet, the results showed no effect at 6 or 12 months. However, the review reported significantly lower hospital readmissions at 6 months (RD -0.05, − 0.09, − 0.00), 12 months (RD -0.11, − 0.17, − 0.05), and at 24 months (MD -1.03, − 1.81, − 0.24), but with no effect after 1 month [32]. One review reported lower readmissions, shorter time to readmission and less hospital days. It also found an inconclusive effect on costs related to the use of emergency departments [31].

### Patient navigator interventions for various chronic diseases and multimorbidity

Patient navigator interventions covering chronic conditions other than cancer and multimorbidity were subject of two systematic reviews (see Table 4: [12, 33]). Similar to the other settings, most interventions focussed on overcoming barriers including appointment scheduling, reminders, patient education, social and emotional support or liaison with providers.

**Table 4:** Patient navigator interventions with a focus on various chronic diseases and multimorbidity

*Skill-mix interventions*				*Outcomes*		
*Details of the intervention*	*Profession(s)*	*Population*	*Countries*	*Patient-related outcomes*	*Health-system related outcomes*	*Source*
Patient navigation interventions addressing patients with one or multiple chronic conditions (e.g. appointment scheduling, reminders, mobilize social support, education, liaison with providers)	Intervention: Lay persons, peers, professional workers Comparison: Not reported	Patients with diabetes, CVD, HIV/AIDS, CKD, dementia, or multiple (including socially disadvantaged patients)	US, CA, ZA	• Improved clinical patient outcomes • Improved mortality rates for HIV patients (non-significant)	•	• [12]^a^
Patient navigation intervention to address barriers in chronic care (e.g. education, referrals, social and emotional support, supporting self-management)	Intervention: case managers, community health workers, public health nurses, health coaches Comparison: Not reported	Patients with diabetes, HIV, kidney failure (mostly from vulnerable communities)	US, ZA	• Improved adherence to recommended care or visits • Improvements in disease-specific patient outcomes	• Fewer hospitalizations and ED visits for patients with diabetes	• [33]

^a^systematic review covering various patient navigator interventions with focus on different diseases and therefore listed in three tables; *CKD* chronic kidney disease, *CVD* cardiovascular disease, *ED* Emergency Department, *HIV/AIDS* human immunodeficiency virus/acquired immunodeficiency syndrome; Country abbreviations: *CA* Canada, *US* United States of America, *ZA* South Africa

#### Professions

Both systematic reviews report on covering programs that employ lay and professional health workers as patient navigators [12, 33].

#### Population

The interventions were mostly targeted at vulnerable or socially disadvantaged population groups and covered chronic conditions such as diabetes, cardio vascular diseases, HIV/AIDS, dementia, chronic kidney disease, kidney failure or multiple chronic diseases [12, 33].

#### Outcomes

The outcomes reported by the systematic reviews were mainly patient-related. Reduced mortality rates among HIV patients [12], improved clinical outcomes for individual diseases [12, 33] and a positive effect on adherence to recommended care [33] were shown. Health system-related outcomes included positive effects on hospitalization rates and emergency department visits for patients with diabetes [12, 33].

## Discussion

This study identified 11 systematic reviews that assessed the impact of patient navigators on patient and health system related outcomes. While intervention components and quality of the included studies varied, the systematic reviews overall show that patient navigation interventions have positive effects on facilitating and improving access to screening and treatment. This was particularly the case for medically underserved and ethnic minority patients in the cancer care setting. Moreover, there is emerging evidence on the effect of patient navigators for other chronic conditions and multimorbidity. In particular, patient navigator interventions in transitional care showed positive effects for patients with chronic conditions, and may result in lower rates of hospital re-admissions. However, the evidence on the latter remains scarce and not consistent over time.

The included reviews particularly demonstrated improvements on health system related measures, namely increased access [15, 30] and reduced waiting times for underserved and ethnic minority patients [11]. Patient navigators helped improve access by reducing barriers created by language, culture and low health literacy, thereby helping ensure a more effective patient pathway and reducing delays in diagnosis and treatment [28, 30]. The findings are consistent with previous studies documenting the positive impact of patient navigators on reducing health disparities [34–38]. Improving access to healthcare services for patients by reducing various barriers ranging from financial and insurance issues, complex paperwork, lack of neighbourhood resources, language barriers, insufficient transportation and childcare can empower and foster trust among underserved patients and communities [34].

An important limitation is that most of the available evidence showing that patient navigation interventions can increase access to healthcare services comes from the US. This may be due to different levels of implementation in other countries, with some of the patient navigator programs outside of the US still being in their piloting and evaluation phase. This limits the generalizability of these findings to other country contexts.

The majority of included systematic reviews focused on cancer care. While the effect of patient navigators in cancer care has been well documented, our findings show that patient navigators are increasingly being implemented for various other chronic conditions [12, 31–33]. Particularly in transitional care, patient navigators can improve patients’ health outcomes and readmission rates for older people with at least one chronic condition [31, 32]. Previous studies reported similar positive effects of patient navigation, particularly for older patient groups who have difficulties to navigate through fragmented health and social care systems [39]. The overall positive impact on post-discharge outcomes for older chronically ill patients [31, 32] with no effect after 1 month [32] is in line with other evidence showing inconsistent effects on readmission rates over time. Considering a threshold of more than 30 days may indicate a positive impact over a prolonged period in time [39, 40].

An important challenge for the evaluation of patient navigation is that models are very different in terms of navigation services offered. There is a lack of knowledge which patient navigator tasks (e.g. education, appointment scheduling, reminders, home visits) are most promising and what is needed to successfully implement the new roles. Moreover, the evidence shows that patients are being navigated through the healthcare system by a variety of different people, ranging from qualified professionals within the health systems such as physicians, nurses, social workers and lay people [41]. However, a detailed description of the educational background, length of training and knowledge about the minimum level of qualification to undertake patient navigator roles is largely missing in the included studies.

## Conclusion

Introducing patient navigator roles can be a strategy to improve access to healthcare services, as shown for cancer patients from ethnic minority or socially disadvantaged population groups. It may also improve patients’ health outcomes and lower readmission rates for patients with other chronic conditions and multimorbidity. More research is needed on the impact of patient navigators outside the US and for chronic conditions other than cancer. Barriers and enabling factors for the successful implementation of patient navigators are to be further investigated. This relates to the definition of scope of practice and the effectiveness of supervision, qualification and skills of patient navigators and how the patient navigator role should be tailored for different patients and population groups. The literature shows that patient navigators mostly collaborate with other providers and professionals. Therefore, further knowledge is also needed on how patient navigators should be integrated into primary care teams.

## Supplementary Information

**Additional file 1.**

## Data Availability

The datasets used and/or analysed during the current study are available from the corresponding author on reasonable request.

## Footnotes

1 kappa coefficient was interpreted as follows: weak: .21–.40; moderate agreement: .41–.60; substantial: .61–.80; almost perfect agreement: .81–.99, based on (Viera & Garrett 2005, http://www1.cs.columbia.edu/~julia/courses/CS6998/Interrater_agreement.Kappa_statistic.pdf)

## References

[1] OECD (2019). Health at a glance: Europe 2018: state of health in the EU cycle.

[2] Nolte E, Knai C (2015). Assessing chronic disease management in European health systems. Country reports.

[3] Notle E, Knai C, Saltman R (2014). Assessing chronic disease management in European health systems. Concepts and approaches.

[4] Roland M, Nolte E (2014). The future shape of primary care. Br J Gen Pract.

[5] Ham C (2009). The ten characteristics of the high-performing chronic care system.

[6] Levesque J, Harris M, Russell G (2013). Patient-centred access to health care: conceptualizing access at the interface of health systems and populations. Int J Equity Health.

[7] Sibbald B, Shen J, McBride A (2004). Changing the skill-mix of the health care workforce. J Health Serv Res Policy.

[8] Freeman H (2012). The origin, evoluation, and principles of patient navigation. Cancer Epidemiol Biomark Prev.

[9] Wells KJ, Battaglia TA, Dudley DJ, Garcia R, Greene A, Calhoun E, Mandelblatt JS, Paskett ED, Raich PC (2008). Patient navigation: state of the art or is it science?. Cancer.

[10] Battaglia T, Roloff K, Posner M, Freund K (2007). Improving follow-up to abnormal breast cancer screening in an urban population: a patient navigation intervention. Cancer.

[11] Robinson-White S, Conroy B, Slavish K, Rosenzweig M (2010). Patient navigation in breast cancer: a systematic review. Cancer Nurs.

[12] McBrien KA, Ivers N, Barnieh L, Bailey JJ, Lorenzetti DL, Nicholas D, Tonelli M, Hemmelgarn B, Lewanczuk R, Edwards A, Braun T, Manns B (2018). Patient navigators for people with chronic disease: a systematic review. PLoS One.

[13] Corbella X, Barreto V, Bassetti S, Bivol M, Castellino P, de Kruijf E, Hojs R (2018). Hospital ambulatory medicine: a leading strategy for internal medicine in Europe. Eur J Intern Med.

[14] Braun KL, Kagawa-Singer M, Holden AE, Burhansstipanov L, Tran JH, Seals BF, Corbie-Smith G, Tsark JU, Harjo L, Foo MA, Ramirez AG (2012). Cancer patient navigator tasks across the Cancer care continuum. J Health Care Poor Underserved.

[15] Ali-Faisal S, Colella T, Medina-Jaudes N, Benz Scott L (2017). The effectiveness of patient navigation to improve healthcare utilization outcomes: a meta-analysis of randomized controlled trials. Patient Educ Couns.

[16] Glick S, Clarke A, Blanchard A, Whitaker A (2012). Cervical cancer screening diagnosis and treatment interventions for racial and ethnic minorities: a systematic review. J Gen Intern Med.

[17] Kline RM, Rocque GB, Rohan EA, Blackley KA, Cantril CA, Pratt-Chapman ML, Burris HA, Shulman LN (2019). Patient navigation in Cancer: the business case to support clinical needs. J Oncol Pract.

[18] Waisel D (2013). Vulnerable populations in healthcare. Curr Opin Anaesthesiol.

[19] Cadzow R, Craig M, Rowe J, Khan L (2013). Transforming community members into diabetes cultural health brokers: the neighborhood health talker project. Diabetes Educ.

[20] Torres S, Spitzer D, Labonté R, Amaratunga C, Andrew C (2013). Community health workers in Canada: innovative approaches to health promotion outreach and community development among immigrant and refugee populations. J Ambul Care Manage.

[21] Ranaghan C, Boyle K, Meehan M, Moustapha S, Fraser P, Concert C (2016). Effectiveness of a patient navigator on patient satisfaction in adults patients in an ambulatory care setting. JBI Database Syst Rev Implement.

[22] Maier C, Kroezen M, Hartl K, Winkelmann J, Wismar M, Busse R (2018). Overview of systematic reviews: outcomes of health workforce skill-mix changes in primary and ambulatory care.

[23] Light R (1971). Measures of response agreement for qualitative data: some generalizations and alternatives. Psychol Bull.

[24] Davies M, Fleiss J (1982). Measuring agreement for multinomial data. Biometrics.

[25] Shea BJ, Grimshaw JM, Wells GA, Boers M, Andersson N, Hamel C, et al. Development of AMSTAR: a measurement tool to assess the methodological quality of systematic reviews. BMC Med Res Methodol. 2007;7(1). 10.1186/1471-2288-7-10. 10.1186/1471-2288-7-10 PMC1810543 17302989

[26] Thomson D, Russell K, Becker L, Klassen T, Hartling L (2010). The evolution of a new publication type: steps and challenges of producing overview of reviews. Res Synth Methods.

[27] Shea, B. J., Reeves, B. C., Wells, G., Thuku, M., Hamel, C., Moran, J., Moher, D., Tugwell, P., Welch, V., Kristjansson, E., & Henry, D. A. (2017). AMSTAR 2: a critical appraisal tool for systematic reviews that include randomised or non-randomised studies of healthcare interventions, or both. BMJ, j4008. 10.1136/bmj.j4008 PMC5833365 28935701

[28] Bush M, Kaufman M, Shackleford T (2017). Adherence in the Cancer care setting: a systematic review of patient navigation to traverse barriers. J Cancer Educ.

[29] Roland K, Milliken E, Rohan E, DeGroff A, White S, Melillo S (2017). Use of community health workers and patient navigators to improve Cancer outcomes among patients served by federally qualified health centers: a systematic review. Health Equity.

[30] Genoff M, Zaballa A, Gany F, Gonzalez J, Ramirez J, Jewell S, Diamond L (2016). Navigating language barriers: a systematic review of patient Navigators' impact on Cancer screening for limited English Profient patients. J Gen Intern Med.

[31] Manderson B, Mcmurray J, Piraino E, Stolee P (2011). Navigation roles support chronically ill older adults through healthcare transitions: a systematic review of the literature. Health Soc Care Commun.

[32] Le Berre M, Maimon G, Sourial N, Guériton M, Vedel I (2017). Impact of transitional Care Services for Chronically ill Older Patients: a systematic evidence review. J Am Geriatr Soc.

[33] Desveaux L, McBrien K, Barnieh L, Ivers NM (2019). Mapping variation in intervention design: a systematic review to develop a program theory for patient navigator programs. Syst Rev.

[34] Natale-Pereira A, Enard K, Nevarez L, Jones L (2011). The role of patient navigators in eliminating health disparities. Cancer.

[35] Ferrante J, Wu J, Dicicco-Bloom B (2011). Strategies used and challenges faced by a breast cancer patient navigator in an urban underserved community. J Natl Med Addoc.

[36] Ellis L, Canchola A, Spiegel D, Ladabaum U, Haile R, Gomez S (2018). Racial and ethnic disparities in Cancer survival: the contribution of tumor, sociodemographic, institutional and neighborhood characteristics. J Clin Oncol Off J Am Soc Clin Oncol.

[37] Yedjou C, Tchounwou P, Payton M, Miele L, Fonseca D, Lowe L, Alo R (2017). Assessing the racial and ethnic disparities in brast cancer mortality in the United States. Int J Environ Res Public Health.

[38] Brennan M, Gormally J, Butow P, Boyle F, Spillane A (2014). Survivorship care plans in cancer: a systematic review of care plan outcomes. Br J Cancer.

[39] Balaban R, Zhang F, Vialle-Valentin C, Galbraith A, Burns M, Larochelle M, Ross-Degnan D (2017). Impact of a patient navigator program on hospital-based and outpatient utilization over 180 days in a safety-net health system. J Gen Intern Med.

[40] Balaban R, Galbraith A, Burns M, Vialle-Valentin C, Larochelle M, Ross-Degnan D. A patient navigator intervention to reduce hospital readmissions among high-risk safety-net patients: a rondomized controlled trial. J Gen Intern Med. 2015;30(7):907–15. 10.1007/s11606-015-3185-x. 10.1007/s11606-015-3185-x PMC4471016 25617166

[41] Carter NV, Feather J, Nicholl J, Cleghorn L (2018). Navigation delivery models and roles of navigators in primary care: a scoping literature review. BMC Health Serv Res.

